# Reliability and validity of a mobile application for femoral anteversion measurement in adult patients

**DOI:** 10.1186/s13018-023-03853-y

**Published:** 2023-05-19

**Authors:** Joon Woo Lee, Minjoon Oh, Mi Na Choi, Seung Yeol Lee

**Affiliations:** Department of Orthopaedic Surgery, Myongji Hospital, Hanyang University College of Medicine, 55, Hwasu-Ro 14Beon-Gil, Deogyang-Gu, Goyang-Si, 10475 Gyeonggi-Do Korea

**Keywords:** Femoral anteversion, Conventional tomography, Mobile application, 3-Dimensional reconstruction, Simple radiograph

## Abstract

**Background:**

Femoral torsion is primarily measured by computed tomography (CT), which has cost and radiation exposure concerns. Recently, femoral anteversion measurement by a simple radiograph-based mobile application was developed for patients with cerebral palsy. This study aimed to validate the use of a mobile application that can reconstruct a three-dimensional model of the femur from conventional radiographs for adults.

**Methods:**

Medical records of 76 patients undergoing conventional femur anteroposterior/lateral radiography and femur CT were reviewed. To measure femoral anteversion *on the reconstructed 3-dimensional images from both the* mobile application and CT, we drew a line which connects the posterior margins of each femoral condyle and another line which passes through the center of the femoral head and the midpoint of the femoral neck. *After the reliability test, a single examiner measured femoral anteversion on the mobile application and CT.* Pearson’s correlation analysis was used to assess the correlation between anteversion on the mobile application and CT.

**Results:**

Femoral anteversion measured on both CT and the mobile application showed excellent reliability (intraclass correlation coefficients: 0.808–0.910). The correlation coefficient between femoral anteversion measured using CT and the mobile application was 0.933 (*p* < 0.001). The correlation of femoral anteversion between CT and the mobile application was relatively higher in the absence of metallic implants (correlation coefficient: 0.963, *p* < 0.001) than in the presence of metallic implants (correlation coefficient: 0.878, *p* < 0.001).

**Conclusions:**

Using two simple radiographs, the mobile application showed excellent validity and reliability for femoral anteversion measurement in adults as compared to CT. With the high accessibility and cost-effectiveness of this mobile application, femoral torsion measurement might be easily performed with simple radiography in clinical settings in the near future.

**Supplementary Information:**

The online version contains supplementary material available at 10.1186/s13018-023-03853-y.

## Background

**Fig. 1:**
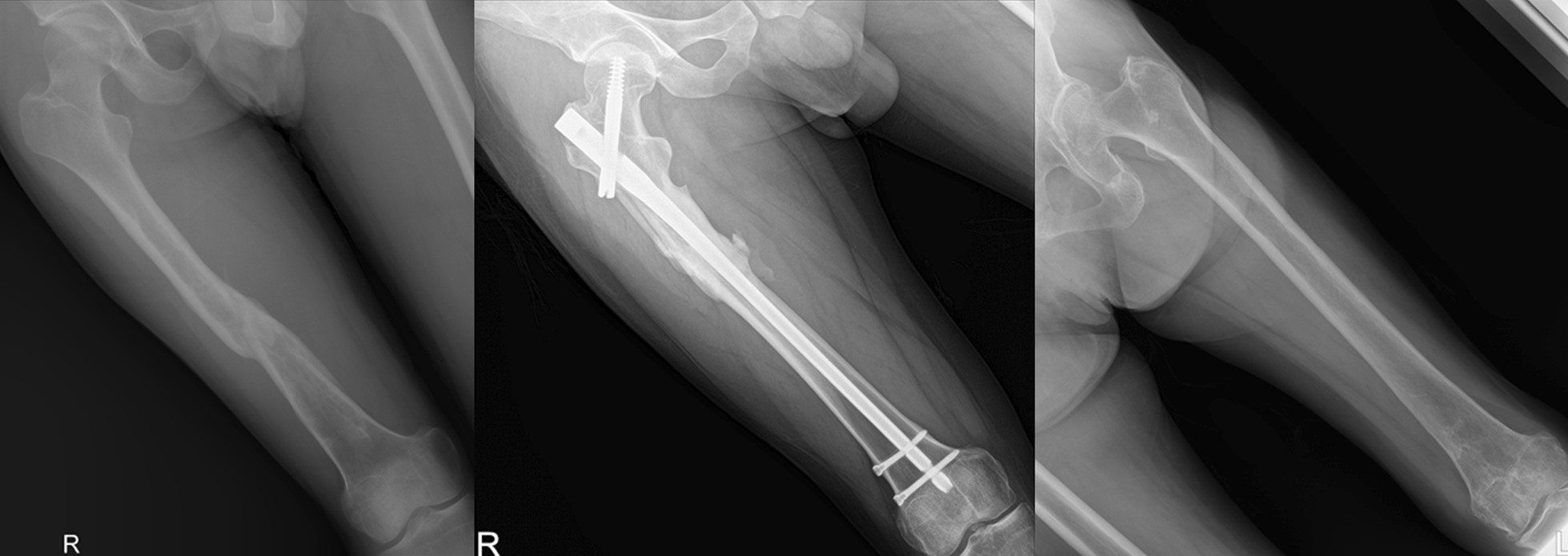
Cases requiring the evaluation of rotational deformity after femoral fractures. Although each fracture was successfully treated and healed, it was difficult to assess rotational deformity without CT imaging

Femoral shaft fractures are high-energy injuries that are frequently encountered in orthopedic departments [1]. Despite substantial improvements in surgical techniques for longitudinal malunion after femoral fractures, rotational malalignment remains a challenge. Femoral malrotation results in pain and loss of motion in the adjacent joints, which can restrict daily life functional activities, such as climbing stairs or jogging [2]. Furthermore, some studies reported a significant correlation between femoral torsion and osteoarthritis of the knee and hip joints [3, 4]. Therefore, accurate femoral torsion measurement is necessary for preoperative planning and postoperative assessment (Fig. 1).

Femur torsion is the angle of femoral anteversion, which is primarily measured by computed tomography (CT) and can also be evaluated with magnetic resonance (MR). While the benefits of CT outweigh radiation exposure risk, radiologists should be aware of its potential harmful effects and avoid unnecessarily high CT doses. Due to cost and radiation exposure concerns, postoperative CT for fractures are not mandatory [5, 6]. Moreover, while femoral torsion might be measured intraoperatively with a modified fluoroscopic C-arm, this method has not been validated for femoral torsion assessment [7].

Recently, femoral anteversion measurement using a simple radiograph-based mobile application showed excellent validity and reliability in patients with cerebral palsy (CP) [8]. This proposed mobile application can be used with conventional X-rays, without any additional equipment required. Therefore, processed biplanar X-rays can be a good alternative to CT and are advantageous in studies of torsional problems in bony structures and rotational problems in articulations with low radiation doses [9]. However, this mobile application was previously used only in young CP patients and has not yet been evaluated in general adults or in patients with inserted implants [8]. CP patients exhibit various femoral deformities, such as coxa valga, coxa vara, and changes in rotational profile, depending on disease severity [10–12]. Therefore, a mobile application developed on the basis of CP patient data is expected to be sufficiently applicable for general adults. As such, this study aimed to validate the use of a mobile application that can reconstruct a three-dimensional (3D) model of the femur from conventional radiographs for adults.

## Methods

This retrospective study (level of evidence: diagnostic level III) was approved by the Institutional Review Board of our hospital (IRB no. MJH-2020-12-020). Due to the retrospective nature of the study, the requirement for informed consent was waived.

Medical records of patients, who underwent conventional femur anteroposterior (AP) and lateral (LAT) radiography and femur CT between May 2010 and April 2021, were reviewed. Conventional radiography and CT were conducted within 3 months from each other. Conventional femur AP and LAT radiographs were obtained by a DK X-ray machine (Samsung, Seoul, South Korea) at a source-to-image distance of approximately 120 cm. The machine was set at 75 kVp and 16 mAs. CT was performed using the Light speed VCT Brilliance 64 (GE Healthcare, Milwaukee, WI, USA) with 120 kVP and 5.0-mm (axial view) and 2.0-mm (coronal, sagittal view) slice thicknesses for the femur. We used a picture archiving and communication system (PACS; Infinitt, Seoul, South Korea) to manipulate and measure the radiographs and then used the Femora® Mobile Application (Didim, Seongnam, Gyeonggi, South Korea) to reconstruct a 3D model from the conventional femur AP and LAT radiographs. The exclusion criteria were as follows: (1) comorbidities affecting skeletal appearance, such as infection and tumor; (2) inadequate radiographs available for review, such as definite femoral deformities; and (3) radiographs or CT images that were not taken at our hospital.

The mobile application was operated on a fourth-generation iPad Air (Apple, Cupertino, CA). Images of the conventional femur AP and LAT radiographs were obtained with an embedded camera, and the touch interface of the device was used for contouring the femur [8]. Once the mobile application-based reconstruction was completed, femoral anteversion was measured with the embedded tool (Additional file 1). To measure femoral anteversion on the reconstructed 3D images from both the mobile application and CT, we drew a line which connects the posterior margins of each femoral condyle and another line which passes through the center of the femoral head and the midpoint of the femoral neck [8].

This study was performed by three investigators to assess the reliability of the mobile application. The examiners independently reconstructed the femur from conventional AP and LAT radiographs using a mobile application. For interobserver reliability, the three examiners measured the femoral anteversion on the reconstructed 3D images from the mobile application and CT. For intraobserver reliability, one examiner (JWL) re-measured the femoral anteversion at a 4-week interval. To assess the inter- and intraobserver reliability, we calculated the interclass correlation coefficient (ICC).

### Statistical analysis

We used ICCs and a two-way random effect model to assess reliability, assuming a single measurement and absolute agreement [13]. Using an ICC target value of 0.8, the Bonett’s approximation was used to set 0.2 as the width of the 95% confidence intervals. The minimum sample size for reliability testing was 36 [14]. ICC values > 0.8 were considered as those with excellent reliability. In bilateral cases, only the right side was analyzed to ensure statistical independence [15]. The Kolmogorov–Smirnov test was used to verify the normality of the distribution of continuous variables. Correlations between two variables were assessed using Pearson’s correlation analysis as the parametric method. Lin's concordance correlation coefficient was added for the agreement analysis [16]. All statistical analyses were conducted using SPSS version 20.0 (IBM Co., Chicago, IL, USA). Statistical significance was set at *p* < 0.05.

## Results

A total of 94 patients met the inclusion criteria. After examining the exclusion criteria, 76 patients were finally included. Eighteen patients were excluded owing to chronic osteomyelitis with deformity (3 patients), bone tumors (2 patients), femoral condyle fracture (6 patients), and outside CT images that were not obtained at our hospital (7 patients). The patients’ mean age at the time of examination was 45.2 ± 21.7 years (range: 20.0–91.0) (Table 1). Forty-two patients underwent radiographic examination due to trauma, and 12 patients had CP and limb deformities (Table 1).

**Table 1:** Patients’ demographics

Parameter	Value
Age (years ± SD)	45.2 ± 21.7 (range 20–91)
Sex (M/F)	26/50
Laterality (right/left)	41/35
Patients with metallic implant	37
The cause of the examination	
Trauma	42
Infection	5
Cerebral palsy	12
Deformity	12
Degenerative disease	4
Soft tissue tumor	1

*SD* standard deviation, *M* male, *F* female

Femoral anteversion measured on both CT and the mobile application showed excellent reliability. Femoral anteversion on the mobile application showed a higher ICC value (0.910 vs. 0.884) for intraobserver reliability and a lower ICC value (0.808 vs. 0.907) for interobserver reliability than CT (Table 2).

**Table 2:** Intra- and interobserver reliability of femoral anteversion

Measurements	Intraobserver reliability		Interobserver reliability	
	ICC	95% CI	ICC	95% CI
CT	0.884	0.789–0.939	0.907	0.846–0.948
Mobile application	0.910	0.804–0.956	0.808	0.649–0.898

**Fig. 2:**
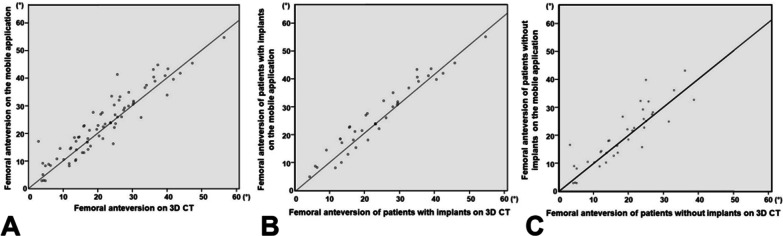
Correlation between femoral anteversion measurements on the mobile application and three-dimensional computed tomography (3D-CT). **A** Overall correlation. **B** Correlation in patients with metallic implant. **C** The correlation in patients without metallic implant

The correlation coefficient between femoral anteversion measured using CT and the mobile application was 0.933 (*p* < 0.001) (Fig. 2). There was no significant difference in femoral anteversion between CT and the mobile application (Table 3). The correlation between CT and the mobile application was affected by the metallic implant in the femur. The correlation of femoral anteversion between CT and mobile application was relatively higher in the absence of metallic implants (correlation coefficient: 0.963, *p* < 0.001) than in the presence of metallic implants (correlation coefficient: 0.878, *p* < 0.001) (Fig. 1). The measurement agreement was also higher in the absence of metallic implants (Table 4).

**Table 3:** Comparison of femoral anteversion measurement between three-dimensional computed tomography (3D-CT) and the mobile application

	Femoral anteversion		Mean difference	95% CI	*p* value
	CT	Femora			
Total (n = 76)	20.3 ± 12.1	24.1 ± 12.4	− 3.9	− 7.8 to 0.0	0.052
Metallic implant group (n = 37)	18.7 ± 9.7	20.4 ± 10.6	− 1.6	− 6.3 to 3.0	0.485
No metallic implant group (n = 39)	24.7 ± 12.2	26.3 ± 12.0	− 1.6	− 7.0 to 3.8	0.557

*CT* computed tomography, *CI* confidence interval

**Table 4:** Agreement of femoral anteversion measurement between three-dimensional computed tomography (3D-CT) and the mobile application

	Lin's concordance correlation coefficient	95% CI
Total	0.924	0.884–0.951
Metallic implant group	0.862	0.756–0.925
No metallic implant group	0.955	0.917–0.975

*CT* computed tomography, *CI* confidence interval

## Discission

Femoral torsion is mainly measured by CT scans. However, concerns regarding the cost, radiation exposure, and limited accessibility remain. Therefore, we performed this study to validate a mobile application that can reconstruct a 3D model of the femur from conventional X-ray images and to determine whether this mobile application is comparable or superior to CT. Femoral anteversion measurement with this mobile application showed excellent reliability and validity in adults. Moreover, the mobile application could measure femoral anteversion in patients with metallic implants.

Considering the radiation exposure of CT, an alternative diagnostic tool has been introduced. Low-dose biplanar X-ray technology (EOS; EOS Imaging) is known for its low radiation exposure as compared with CT [17] as well as its validity and reliability for femoral anteversion measurement [18–20]. However, the EOS system also has some disadvantages, such as high cost and limited accessibility. To overcome these limitations in femoral anteversion measurement, the mobile application Femora® was developed. Femora® previously showed excellent validity in femoral anteversion measurement [8]. Additionally, if the mobile application in adults and patients with metallic internal fixation is validated, femoral torsion could be evaluated without postoperative CT. As such, it would be useful in patients with comminuted femoral shaft fractures and those with metal implants around the joint, which would induce noise on CT images. In addition to trauma patients, patients with gait disturbances, such as in-toeing, can simply measure femoral torsion in outpatient clinics with this mobile application.

Femora® was developed based on data of pediatric patients with CP [8] who have a wider spectrum of femoral deformities, including torsional deformity, than those without CP [21]. Considering this database of Femora®, this mobile application can certainly be used for adults with acquired torsional deformity of the femur. As we hypothesized, measuring femoral anteversion with the mobile application showed excellent concurrent validity as compared to CT, the gold standard for femoral anteversion measurement. This finding suggested that the mobile application can be used for adult patients, particularly in postoperative evaluation of femoral torsion.

**Fig. 3:**
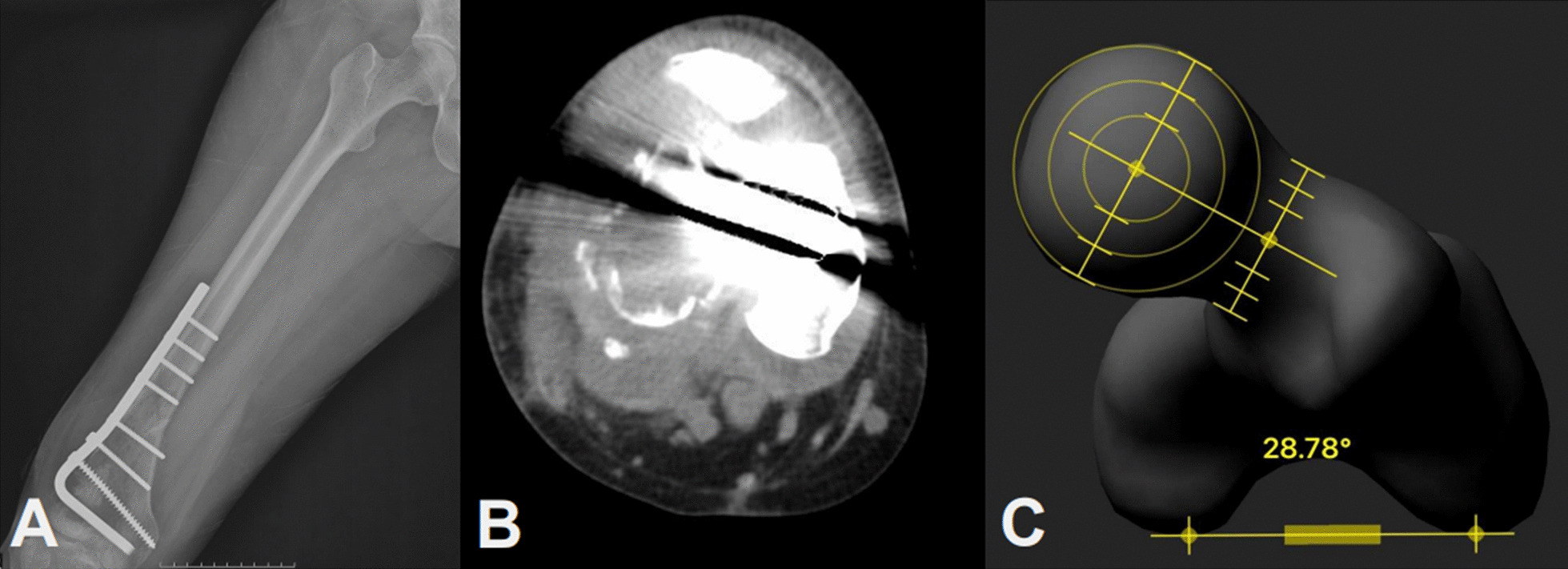
A 52-year-old female patient who underwent operation for a distal femur fracture. **A** Postoperative simple radiograph. **B** Rendering noise in the computed tomography (CT) image. **C** A three-dimensional (3D) model from the conventional femur anteroposterior (AP) and lateral (LAT) radiographs generated by the mobile application

During concurrent validity assessment of the mobile application, the correlation between CT and the mobile application was affected by the metallic implant on the femur. In patients with metallic implants, the noise created by the metallic implants hindered the investigators from measuring femoral anteversion (Fig. 3A, B). To overcome this obstacle, we divided our subjects into two groups (with or without metallic implants) to analyze the validity of the Femora® application. The noise caused a lower correlation between Femora® and CT in the metallic implant group, as compared to the non-metallic implant group. There may be concerns regarding decreased validity of the mobile application for femoral torsion measurement in patients with metallic implants. However, the noise was worse in CT scans, which might have worsened the decrease in correlation coefficient (Fig. 2). When the metallic implants were inserted at the femoral neck or condyles, it was difficult to anatomically locate the femur for femoral anteversion measurement because of the noise in CT scans. However, the noise did not interrupt femoral anteversion measurement on the reconstructed images by Femora® (Fig. 3C). The agreement analysis showed that the no-metallic group had a relatively higher concordance correlation coefficient, indicating that metallic implants may affect the measurement of femoral anteversion on CT images. Therefore, we concluded that the mobile application might have an advantage for patients with metallic implants as compared to conventional CT for femoral torsion.

This study had some limitations. First, it was a retrospective study. As part of the analysis, medical records were carefully reviewed, and patients with factors that could affect the anatomical deformity were excluded. However, we did not control for some patient factors, including *sex, medical history,* fracture, implant type, and position. As such, a well-designed prospective study is required for mobile applications to be widely utilized. Second, the sample size was relatively small. Although the number of patients in this study was larger than the minimum sample size required for reliability testing, validity verification with a large sample size will allow this mobile application to respond well to various potential deformities and femur fracture patterns.

## Conclusion

Compared to CT, the femoral mobile application using two simple radiographs showed excellent validity and reliability for femoral anteversion measurement in adults. Due to the high accessibility and cost-effectiveness of this mobile application, torsional measurement of the femur might be easily performed with simple radiography in clinical settings in the near future.

## Supplementary Information

**Additional file 1**. Femora® demonstration video.

## Data Availability

The datasets generated during and analyzed during the current study are not publicly available due to their containing information that could compromise the privacy of research participants but are available from the corresponding author upon reasonable request.

## Abbreviations

3D: Three-dimensional
AP: Anteroposterior
CP: Cerebral palsy
CT: Computed tomography
ICCs: Intraclass correlation coefficients
LAT: Lateral

## References

[1] Lindsey JD, Krieg JC (2011). Femoral malrotation following intramedullary nail fixation. J Am Acad Orthop Surg.

[2] Bråten M, Terjesen T, Rossvoll I (1995). Femoral shaft fractures treated by intramedullary nailing. A follow-up study focusing on problems related to the method. Injury.

[3] Eckhoff DG, Kramer RC, Alongi CA, VanGerven DP (1994). Femoral anteversion and arthritis of the knee. J Pediatr Orthop.

[4] Tönnis D, Heinecke A (1991). Diminished femoral antetorsion syndrome: a cause of pain and osteoarthritis. J Pediatr Orthop.

[5] The 2007 recommendations of the International Commission on Radiological Protection. ICRP publication 103. Ann ICRP. 2007. ICRP Publication;37:1–332. 10.1016/j.icrp.2007.10.003 18082557

[6] Wrixon AD (2008). New ICRP recommendations. J Radiol Prot.

[7] Keast-Butler O, Lutz MJ, Angelini M, Lash N, Pearce D, Crookshank M (2012). Computer navigation in the reduction and fixation of femoral shaft fractures: a randomized control study. Injury.

[8] Sung KH, Youn K, Chung CY, Kitta MI, Kumara HC, Min JJ (2020). Development and validation of a mobile application for measuring femoral anteversion in patients with cerebral palsy. J Pediatr Orthop.

[9] Chaibi Y, Cresson T, Aubert B, Hausselle J, Neyret P, Hauger O (2012). Fast 3D reconstruction of the lower limb using a parametric model and statistical inferences and clinical measurements calculation from biplanar X-rays. Comput Methods Biomech Biomed Eng.

[10] Kwon DG, Lee SY, Kim TW, Chung CY, Lee KM, Sung KH (2013). Short-term effects of proximal femoral derotation osteotomy on kinematics in ambulatory patients with spastic diplegia. J Pediatr Orthop B.

[11] Lee KM, Chung CY, Sung KH, Kim TW, Lee SY, Park MS (2013). Femoral anteversion and tibial torsion only explain 25% of variance in regression analysis of foot progression angle in children with diplegic cerebral palsy. J Neuroeng Rehabil.

[12] Sung KH, Kwon SS, Chung CY, Lee KM, Kim J, Lee SY (2018). Fate of stable hips after prophylactic femoral varization osteotomy in patients with cerebral palsy. BMC Musculoskelet Disord.

[13] Shrout PE, Fleiss JL (1979). Intraclass correlations: uses in assessing rater reliability. Psychol Bull.

[14] Bonett DG (2002). Sample size requirements for estimating intraclass correlations with desired precision. Stat Med.

[15] Park MS, Kim SJ, Chung CY, Choi IH, Lee SH, Lee KM (2010). Statistical consideration for bilateral cases in orthopaedic research. J Bone Joint Surg Am.

[16] Lin LI (1989). A concordance correlation coefficient to evaluate reproducibility. Biometrics.

[17] Folinais D, Thelen P, Delin C, Radier C, Catonne Y, Lazennec JY (2013). Measuring femoral and rotational alignment: EOS system versus computed tomography. Orthop Traumatol Surg Res.

[18] Buck FM, Guggenberger R, Koch PP, Pfirrmann CW (2012). Femoral and tibial torsion measurements with 3D models based on low-dose biplanar radiographs in comparison with standard CT measurements. AJR Am J Roentgenol.

[19] Pomerantz ML, Glaser D, Doan J, Kumar S, Edmonds EW (2015). Three-dimensional biplanar radiography as a new means of accessing femoral version: a comparitive study of EOS three-dimensional radiography versus computed tomography. Skelet Radiol.

[20] Thépaut M, Brochard S, Leboucher J, Lempereur M, Stindel E, Tissot V (2016). Measuring physiological and pathological femoral anteversion using a biplanar low-dose X-ray system: validity, reliability, and discriminative ability in cerebral palsy. Skelet Radiol.

[21] Rethlefsen SA, Healy BS, Wren TA, Skaggs DL, Kay RM (2006). Causes of intoeing gait in children with cerebral palsy. J Bone Joint Surg Am.

